# Assessment of Antibiotic Resistance and Efflux Pump Gene Expression in Neisseria Gonorrhoeae Isolates from South Africa by Quantitative Real-Time PCR and Regression Analysis

**DOI:** 10.1155/2022/7318325

**Published:** 2022-10-21

**Authors:** Nireshni Mitchev, Ravesh Singh, Veron Ramsuran, Arshad Ismail, Mushal Allam, Stanford Kwenda, Florah Mnyameni, Nigel Garrett, Khine Swe Swe-Han, Abraham J. Niehaus, Koleka P. Mlisana

**Affiliations:** ^1^School of Laboratory Medicine and Medical Sciences, University of KwaZulu Natal (UKZN), Durban, South Africa; ^2^National Health Laboratory Service, Durban, South Africa; ^3^Sequencing Core Facility, National Institute for Communicable Diseases, National Health Laboratory Service, Johannesburg, South Africa; ^4^Department of Biochemistry and Microbiology, Faculty of Science Engineering and Agriculture, University of Venda, Thohoyandou, South Africa; ^5^Department of Genetics and Genomics, United Arab Emirates University, Al Ain, UAE; ^6^Centre for the AIDS Programme of Research in South Africa, Durban, South Africa; ^7^School of Nursing and Public Health, UKZN, Durban, South Africa; ^8^National Health Laboratory Service, Johannesburg, South Africa

## Abstract

**Introduction:**

Treatment of gonorrhoea infection is limited by the increasing prevalence of multidrug-resistant strains. Cost-effective molecular diagnostic tests can guide effective antimicrobial stewardship. The aim of this study was to correlate mRNA expression levels in *Neisseria gonorrhoeae* antibiotic target genes and efflux pump genes to antibiotic resistance in our population.

**Methods:**

This study investigated the expression profile of antibiotic resistance-associated genes (*penA*, *ponA*, *pilQ*, *mtrR*, *mtrA*, *mtrF*, *gyrA*, *parC*, *parE*, *rpsJ*, *16S rRNA*, and *23S rRNA*) and efflux pump genes (*macAB*, *norM,* and *mtrCDE*), by quantitative real-time PCR, in clinical isolates from KwaZulu-Natal, South Africa. Whole-genome sequencing was used to determine the presence or absence of mutations.

**Results:**

*N. gonorrhoeae* isolates, from female and male patients presenting for care at clinics in KwaZulu-Natal, South Africa, were analysed. As determined by binomial regression and ROC analysis, the most significant (*p* ≤ 0.05) markers for resistance prediction in this population, and their cutoff values, were determined to be *mtrC* (*p* = 0.024; cutoff <0.089), *gyrA* (*p* = 0.027; cutoff <0.0518)*, parE* (*p* = 0.036; cutoff <0.0033)*, rpsJ* (*p* = 0.047; cutoff <0.0012), and 23S rRNA (*p* = 0.042; cutoff >7.754).

**Conclusion:**

Antimicrobial stewardship includes exploring options to conserve currently available drugs for gonorrhoea treatment. There is the potential to predict an isolate as either susceptible or nonsusceptible based on the mRNA expression level of specific candidate markers, to inform patient management. This real-time qPCR approach, with few targets, can be further investigated for use as a potentially cost-effective diagnostic tool to detect resistance.

## 1. Introduction

Increasing antimicrobial resistance (AMR) to *Neisseria gonorrhoeae* is now a public health priority [1, 2] as it threatens the current World Health Organization (WHO) recommended dual therapy (ceftriaxone and azithromycin) [1–3]. Molecular mechanisms of drug resistance have been well characterized [4, 5] and are mainly due to mutational alterations of the drug target, plasmids, and efflux pumps [6, 7].

Globally, 87 million new cases of the sexually transmitted infection (STI) gonorrhoea occur annually, where the highest prevalence has been reported in the WHO Africa region [8]. The estimated prevalence of gonorrhoea in African countries was reported to be 1.4%–15.2%, with higher prevalence in high-risk groups (sex-workers and participants recruited from venues considered to have a higher probability for acquiring infection, e.g., bars) [9]. South Africa has an estimated prevalence of ∼5% [9, 10]. *N. gonorrhoeae* infections are usually localized to the mucosal surfaces of the hosts initial exposure to the organism [11, 12]. Infection of the male urethra causes urethritis (inflammation of the urethra), the symptoms of which, include purulent discharge and dysuria [13]. While male urethritis commonly produces symptoms, gonorrhoea in women is often asymptomatic [14]. The sequelae of untreated gonorrhoea includes acute urethritis, cervicitis, pelvic inflammatory disease (PID), infertility, abortion, ectopic pregnancy, maternal death, and neonatal blindness [15–19].

In Africa and other resource-limited settings, syndromic management of patients remains the main STI management model. Syndromic and empiric treatment leads to overtreatment [20] and contributes to the development of resistance to currently recommended drugs in many parts of the world [21–24]. There is no vaccine for gonorrhoea yet; thus, its prevention and control depends on an accurate diagnosis and appropriate antimicrobial therapy [25]. Currently, treatment options are few and antimicrobial stewardship programmes can reduce antibiotic resistance [26]. There is an urgent need for rapid diagnostic tools to direct therapy [27, 28].

The AMR mechanisms via which *N. gonorrhoeae* has developed resistance has been thoroughly reviewed [4, 29]. These include antimicrobial inactivation, alteration of target sites, increased export via efflux pumps, and decreased uptake via porins [29]. Resistance-to-penicillin and extended-spectrum cephalosporins (ESC) have been associated with modifications and recombination within *penA, porB, ponA* [30], and the presence of *bla*_TEM_ plasmid (penicillin) [4, 31]. Modifications in *penA* result in decreased affinity for penicillin, and recombination with *penA* genes from commensal *Neisseria* species has led to the development of mosaic *penA* alleles which causes resistance-to-penicillin, cefixime, and ceftriaxone [4, 32, 33]. The mutation L421P in *ponA* reduces the rate of acylation with penicillin [34]. Mutations in *porB*, which encode porinB, reduce the porin permeability, which then reduces penicillin influx [4]. The *bla*_*TEM*_*-1* gene is responsible for plasmid-mediated resistance-to-penicillin, and a previous study from South Africa showed a prevalence of 66% in nonsusceptible isolates [35]. Mutations in *mtrR*, as well as its promoter region, can cause overexpression of the MtrCDE efflux pump, which has been associated with resistance-to-hydrophobic agents (penicillin, cefixime, ceftriaxone, and azithromycin) [36]. Pore formation in the outer membrane is encoded for by the pilQ gene, mutations in this gene result in reduced antibiotic influx and high-level resistance-to-penicillin [4, 12, 37–39]. When treating patients with an antibiotic, low-level resistance means that an increased dose of the antibiotic can still overcome the resistance to it and clear the infection. High-level resistance, however, means that even an increased dose will not be able to clear the infection i.e., the antibiotic should not be used.

Resistance to tetracycline has been associated with the presence of tetM and mutations in rpsJ, mtrR, and porB [36]. The tetM gene confers high-level plasmid-mediated resistance-to-tetracycline by binding to the 30S ribosomal subunit, thus, releasing the tetracycline molecule and protein synthesis continues [40]. Chromosomally mediated resistance is due to the ribosomal subunit being modified, thus, increasing the efflux and decreasing the influx of tetracycline [4]. The *rpsJ* mutation V57M, alters the binding site, thus, reducing binding affinity of tetracycline for the ribosome [41]. As described for penicillin, modifications in *mtrR* and *porB*, which result in reduced drug accumulation, also contribute to resistance-to-tetracycline [4, 12, 42].

Resistance to ciprofloxacin is due to mutations in *gyrA* and *parC* [36], and mutations in the *norM* promoter results in overexpression of NorM efflux pump, which increases ciprofloxacin MICs [4, 7, 43]. Quinolones inhibit DNA gyrase (encoded by *gyrA* and *gyrB*) and topoisomerase IV (encoded by *parC* and *parE*), which are essential for DNA metabolism, resulting in bactericidal activity [4]. Mutations in these genes alter quinolone recognition of the enzymes and result in resistance [4, 29]. Although many mutations have been identified in *gyrA* and *parC*, the key mutations responsible for quinolone resistance include gyrA_S91F, gyrA_D95N, parC_S88P, and parC_E91K [4, 5, 44]. Mutations in the *gyrB* and *parE* genes did not significantly impact resistance-to-ciprofloxacin. Mutation in the *norM* promoter results in overexpression of the NorM efflux pump, which decreases ciprofloxacin susceptibility MICs [4, 45].

High-level resistance-to-spectinomycin is due to the mutation C1192U in 16S rRNA by reducing target affinity [42]. Resistance-to-azithromycin is often due to mutations in 23S rRNA, namely C2611T (low-level resistance) or A2059 (high-level resistance) [4, 42]. Mutation in the promoter regions of MacAB and *mef*-encoded efflux pumps result in overexpression contributing to resistance-to-macrolides [4, 7, 42, 43, 46].

A range of molecular diagnostic approaches have been evaluated, each with its strengths and limitations [42, 47–51]. While most methods target specific mutations to infer resistance, our approach targets the gene and its expression levels to infer resistance. Genomics prediction tools and equations have been extremely effective in characterizing antimicrobial resistance mechanisms [52, 53]. At present, ResistancePlus® GC (SpeeDx Pty Ltd, Sydney, Australia), which detects resistance-to-ciprofloxacin, is the only commercially available genotypic resistance testing assay for *N. gonorrhoeae*. The assay uses real-time PCR to detect species-specific *porA* and *opa* genes for identification and differentiates the *gyrA*_S91 wild type from the *gyrA*_S91F mutant to determine susceptibility or resistance-to-ciprofloxacin. In populations where the resistance-to-ciprofloxacin is high, assays which predict resistance to additional drugs would be beneficial.

The aim of this study was to correlate mRNA expression levels in *N*. *gonorrhoeae* antibiotic target genes and efflux pump genes to antibiotic resistance in our population using real-time qPCR, a cost-effective alternative to WGS. A secondary objective was to determine from isolates, if any genes are expressed more in either gender.

## 2. Materials and Methods

### 2.1. Source of Isolates

All 110 *N. gonorrhoeae* isolates in this study were stored and analysed at the University of KwaZulu-Natal Department of Medical Microbiology. The specimens were collected between 2013 and 2016 from male and female patients attending KZN public healthcare clinics for STI care during ethics approved studies. Ethical approval for this study was granted by the Biomedical Research Ethics Committee of the University of KwaZulu-Natal BREC/00000097/2019.

### 2.2. Identification of *Neisseria gonorrhoeae*

Stored *N. gonorrhoeae* isolates (vaginal and urethral specimens) were revived on nonselective Thayer Martin media (supplemented with 1% Vitox, excluding antibiotic supplements) for 18–24 hours in a 37°C 5% CO_2_ incubator. Identification was confirmed (supplementary Table 1 and supplementary Figure 1) using bright field microscopy (*N. gonorrhoeae* is a Gram-negative diplococcus), Bactident® Oxidase rapid test (Merck, Germany) (*N. gonorrhoeae* is oxidase positive), and Phadebact® Monoclonal GC test (Pharmacia, Sweden) (a coagglutination technique used for the definitive identification of *N. gonorrhoeae*) [11, 13, 54]. In addition, a real-time PCR assay, *N. gonorrhoeae* TaqMan® probe Ba046466252 (Thermo Scientific) was used for molecular identification. A subset of 61 male and female isolates with similar antibiotic profiles were selected for WGS. This data confirmed the identification of *N. gonorrhoeae* using Kraken [55] and Pathogenwatch [56].

### 2.3. Phenotypic Antibiotic Susceptibility Testing

Antimicrobial susceptibility testing was performed, using Etest® (bioMérieux, Marcy l'Etoile, France), for all isolates, using GC agar base medium (used for the isolation and cultivation of *N. gonorrhoeae*) supplemented with 1% Vitox (Oxoid) [57–59]. The minimum inhibitory concentration (MIC) was determined as the lowest concentration of the drug to visually inhibit the growth of the organism. The drugs and concentration ranges were as follows; penicillin (0.016–256 *μ*g/mL), ciprofloxacin (0.002–32 *μ*g/mL), ceftriaxone (0.002–32 *μ*g/mL), cefixime (0.016–256 *μ*g/mL), spectinomycin (0.064–1024 *μ*g/mL), tetracycline (0.016–256 *μ*g/mL), and azithromycin (0.016–256 *μ*g/mL). Susceptibility was interpreted as per the European Committee on Antimicrobial Susceptibility Testing (EUCAST) guidelines [60]. Nitrocefin (a chromogenic cephalosporin substrate) was used to detect *β*-lactamase production, which indicates resistance to beta-lactam antibiotics such as penicillin [61]. The 2016 WHO gonococcal reference strains (F, G, K, L, M, N, O, P, U, V, W, X, Y, Z) [62] and ATCC 49226 were used as controls in this study.

### 2.4. Whole-Genome Sequencing and Assembly

DNA was extracted using the PureLink™ Microbiome DNA Purification Kit (ThermoFisher Scientific) as per the manufacturer's instructions. Paired-end libraries were prepared using the Nextera DNA Prep kit, followed by sequencing (2 × 75 bp) on a NextSeq platform (Illumina, Inc., USA). Raw paired-end (PE) reads were initially run through the Jekesa pipeline v1.0 [63] for WGS bacterial typing. Briefly, Trim Galore v0.6.2 [64]; was used to filter the PE reads (*Q* > 30 and length >50 bp). *De novo* assembly and polishing of assemblies were performed using SPAdes v.3.13 [65] and Shovill v1.1.0 [66], respectively. Assembly metrics were calculated using QUAST v5.0.2 [67]. AMR markers were identified using PointFinder [68] and confirmed using Pathogenwatch [69] and Clustal Omega [70]. Whole-genome sequence data is available in DDBJ/ENA/GenBank with the BioProject number PRJNA681740.

### 2.5. RNA Extraction and cDNA Synthesis

RNA was extracted from the *N. gonorrhoeae* isolates using TRIzol™ reagent (Invitrogen) with the PureLink™ RNA Mini Kit (ThermoFisher Scientific) and PureLink™ DNase (ThermoFisher Scientific) as per the manufacturer's instructions. The total RNA concentration was quantified using a nanodrop spectrophotometer, and samples were used only if the optical density at 260 nm (OD_260/280_) was ∼2.0. RNA integrity was confirmed using a bleach gel method [71]. One microgram of total RNA from each sample was reversed transcribed using the iScript™ Reverse Transcription Supermix for RT-qPCR (Bio-Rad) as per the manufacturer's instruction and reaction protocol. The total cDNA concentration was quantified, and samples were used only if the optical density at 260 nm (OD_260/280_) was >1.8.

### 2.6. RNA Quantification by Real-Time PCR

Quantitative real-time PCR was performed for the primer sequences listed in Table 1 cDNA was diluted using a 1 : 10 ratio for real-time PCR analysis. Each PCR mixture (5 *μ*l total volume) consisted of the respective primers (0.5 pmol/*μ*l for *farB* and *mtrD*; 0.7 pmol/*μ*l for 16S rRNA, *penA* and *macA*; 0.3 pmol/*μ*l for all other primers), 2.5 *μ*l PowerUp™ SYBR™ Green Master Mix (ThermoFisher Scientific, USA), 1 *μ*g cDNA and nuclease-free water. Reactions were run in duplicate on the Quant Studio 5 (ThermoFisher, CA, USA) (1 cycle at 95°C, 2 min.), followed by 40 cycles consisting of denaturation at 95°C (15 sec.), annealing at 60°C (15 s), extension at 72°C (1 min). Followed by a melt curve stage (95°C, 15°s) ramp rate 1.6°C/s, 60°C (1 min) ramp rate 1.6°C/s, and 95°C (15 s) ramp rate 0.15°C/s. Amplification specificity was confirmed using melting curve analysis and gel electrophoresis. Serial dilutions of cDNA from total RNA (control strain WHO F) were performed for each target. These served as standard curves for quantitative analysis. The Quant Studio 5 (ThermoFisher, CA, USA) analysis software version 3.3 was used for quantitative analysis. The expression levels were calculated after normalization to a housekeeping gene (16S rRNA).

### 2.7. Statistical Analysis

Nonparametric statistical analysis and correlations were performed using GraphPad Prism v5.0 (Graphpad Software Inc. CA, USA) and IBM® SPSS Statistics v27. The differences between the groups (susceptible/nonsusceptible, mutations/no mutations, and males/females) were evaluated using *t*-tests. The regression analysis was performed to determine the relationship of susceptibility as the dependant variable to mRNA levels of antibiotic resistance-associated genes as the independent variables. For each drug, the regression model included all independent variables. A *p* -value <0.05 was considered to denote statistical significance. Using a receiver operating characteristic (ROC) analysis (which assesses the accuracy of model predictions), cutoff values were calculated for genes associated with resistance for each drug.

## 3. Results

Resistance-to-penicillin, tetracycline, and ciprofloxacin were high in our isolates (supplementary Figure 2), also described in our previous paper [35]. All isolates were susceptible to spectinomycin, azithromycin, ceftriaxone, and cefixime. A total of 110 isolates were analysed to determine differences in mRNA levels between susceptible and nonsusceptible isolates. A subset of 61 isolates with similar MIC values (30 vaginal swabs and 31 urethral swabs) with similar MIC values were analysed to determine differences in mRNA levels between males and females and for differences in mRNA levels between isolates with and without resistance-associated mutations.

### 3.1. Comparison of Gene Expression Levels between Susceptible and Nonsusceptible Isolates

We found that for all drugs tested, expression levels between the two groups (susceptible and nonsusceptible) were significantly different (supplementary Table 2). For penicillin (*penA*, *ponA*, *pilQ*, *mtrR*, *mtrC*, *mtrD*, *mtrE*, *mtrA,* and *mtrF*) the *p* values ranged from 0.01–0.04 (except *mtrF*, *p* value 0.17). For ciprofloxacin (*gyrA*, *parC*, *parE,* and *norM*) the *p* values were ≤0.001. For tetracycline (*rpsJ*, *mtrR*, *mtrC*, *mtrD*, *mtrE*, *mtrA,* and *mtrF*) the *p* values ranged from 0.001–0.04. For azithromycin (23S rRNA, *macA*, *macB*, *mtrR*, *mtrC*, *mtrD*, *mtrE*, *mtrA,* and *mtrF*) the *p* values ranged from ≤0.001–0.01 (except *mtrC*, *p* value 0.1). For spectinomycin, the *p* value for 16S rRNA was ≤0.001. For ESC (*penA*, *mtrR*, *mtrC*, *mtrD*, *mtrE*, *mtrA,* and *mtrF*) the *p* values ranged from ≤0.001–0.05.

### 3.2. Comparison of Gene Expression Levels between Isolates with No Mutations Vs. Isolates with Mutations

The median expressions of antimicrobial and efflux pump genes (f*arA*, *farB*, *gyrA*, *macA*, *macB*, *mtrA*, *mtrC*, *mtrD*, *mtrE*, *mtrF*, *mtrR*, *norM*, *parC*, *parE*, *penA*, *ponA*, *pilQ*, *rpsJ*, 16S rRNA, and 23S rRNA) associated with resistance were examined to determine any differences between isolates with mutations in the resistance-associated genes compared to isolates without mutations (supplementary Table 3). We found significant differences between the wildtype genes and isolates genes with mutations *mtrF*_V213I (*p*=0.02), *gyrA*_S91F (*p*=0.02), *gyrA*_D95G (*p*=0.026), *parC*_S87N (*p*=0.023), *parC*_V384I (*p* ≤ 0.001), and *macA*_A8S (*p*=0.01).

### 3.3. Comparison of Gene Expression Levels between Males and Females

The median expressions of antimicrobial target genes and efflux pump genes (*farA*, *farB*, *gyrA*, *macA*, *macB*, *mtrA*, *mtrC*, *mtrD*, *mtrE*, *mtrF*, *mtrR*, *norM*, *parC*, *parE*, *penA*, *ponA*, *pilQ*, *rpsJ*, 16S rRNA, and 23S rRNA) associated with resistance were examined to determine any differences between isolates from males and females. We found no significant difference between the expression levels of isolates from males compared to females. This was confirmed when we subdivided groups into male susceptible, male nonsusceptible, female susceptible, and female nonsusceptible.

### 3.4. Correlation of mRNA Expression Levels with Resistance

*penA*, *ponA*, *pilQ*, *mtrR*, *mtrC*, *mtrD*, *mtrE*, *mtrA*, *mtrF*, *gyrA*, *parC*, *parE* and *norM*, *rpsJ*, 16S, 23S, *macA,* and *macB* mRNA expression levels were determined for an association with resistance status using a logistic regression analysis (Table 2).

Binomial logistic regression was performed to ascertain the effects of *penA*, *ponA*, *pilQ*, *mtrR*, *mtrC*, *mtrD*, *mtrE*, *mtrA,* and *mtrF* on the likelihood that isolates were resistant to penicillin. The model explained 28.6% (Nagelkerke R2) of the variance in resistance-to-penicillin and correctly classified 81.0% of cases. Sensitivity was 96.3% and specificity was 29.2%. Of the predictor variables, *mtrC* was statistically significant (*p*=0.024). The discrimination of this model, as determined by ROC curve analysis, is acceptable (AUC 0.8). The logistic regression model was statistically significant, *p* < 0.009. The regression analysis was used to ascertain the effects of *gyrA*, *parC*, *parE,* and *norM* on the likelihood that isolates were resistant-to-ciprofloxacin. The model explained 19% (Nagelkerke R2) of the variance in resistance-to-ciprofloxacin and correctly classified 65.8% of cases. Sensitivity was 90.1% and specificity was 30.6%. Of the predictor variables, *gyrA* and *parE* were statistically significant, *p*=0.027 and 0.036, respectively. The discrimination of this model, as determined by the ROC curve analysis, is acceptable (AUC 0.7). The logistic regression model was statistically significant, *p*=0.001.

Regression analysis was used to ascertain the effects of 23S rRNA, *macA*, *macB*, *mtrR*, *mtrC*, *mtrD*, *mtrE*, *mtrA*, and *mtrF* on the likelihood that isolates were resistant to azithromycin. The model explained 67.9% (Nagelkerke R2) of the variance in resistance-to-azithromycin and correctly classified 94.9% of cases. Sensitivity was 42.9% and specificity was 98.2%. Of the predictor variables, 23S rRNA was statistically significant (*p*=0.042). The discrimination of this model, as determined by the ROC curve analysis, is outstanding (AUC 0.98). The logistic regression model was statistically significant, *p* ≤ 0.001.

For tetracycline, spectinomycin, and ESC, the discrimination of the models was excellent (AUC >9) and correctly classified >98% of cases. However, due to a low number of data points in either the susceptible or resistant groups, the regression models for these drugs were not statistically significant.

### 3.5. ROC Analysis of qPCR Data

To determine the threshold (cutoff) of individual genes determined as drug-resistant, a ROC (receiver operating characteristic) analysis was performed. Using the AUC, cutoff, sensitivity, and specificity results (listed, respectively), we evaluated the qPCR assays as a tool to predict resistance to each drug (Table 3). Multiple resistance-associated genes for each antibiotic showed high sensitivities (82%–100%). The performance characteristics of the significant markers, as determined by regression analysis, were as follows: *mtrC* (0.63; <0.0890; 84%; 44%), *gyrA* (0.66; <0.0518; 83%; 33%), *parE* (0.67; <0.0033; 88%; 33%), *rpsJ* (0.72; <0.0012; 91%; 33%), 16S rRNA (0.96; <0.0454; 100%; 96%), and 23S rRNA (0.99; >7.754; 86%; 99%).

## 4. Discussion

DNA-based diagnostic approaches which detect resistance-associated single nucleotide polymorphisms (SNPs) are commonly investigated for use in *N. gonorrhoeae* AMR diagnosis. These approaches require the detection of multiple known mutations to infer resistance to a particular drug. In this study, we considered the whole gene rather than SNPs, and investigated the expression of known antibiotic target genes and efflux pump genes and correlated gene expression with AMR. To determine if sex-specific environments contribute to the transcription of AMR genes, we compared expression levels from isolates with similar susceptibility profiles from males and females. Regression analysis was used to determine the strongest predictors of drug resistance in our population, and using a ROC analysis; we estimated cutoff values.

The antibiotic target genes in *N. gonorrhoeae* have been widely described [4, 12]. Alterations in antibiotic target genes are associated with increased MICs and resistance. Our analysis shows that expression levels of antibiotic target genes are significantly higher in susceptible isolates compared to nonsusceptible isolates. A recent RNA-based study identified candidate markers from the transcriptomes which were highly expressed in the susceptible isolates only [51]. The *mtrR* gene, which represses the MtrCDE efflux pump, was higher in the susceptible group. This emphasizes the role of *mtrR* in AMR. MtrR has a more diverse regulatory role than only the MtrCDE efflux pump. It has also been shown to regulate MtrF (inner membrane accessory protein for efficient drug efflux) and FarR (repressor of the farAB efflux pump) [72]. MtrR has also been described to play a role in the expression of two other genes involved in susceptibility, *ponA,* and *pilQ*. By increasing the expression of *ponA* (encodes penicillin binding protein) and repressing the expression of *pilQ* (channel for entry for penicillin) [72].

Mutations can interrupt cellular processes and often hold the key to understanding gene function. Mutations in target genes are associated with resistance; however, for most mutations, we did not see any significant difference in gene expression between isolates with mutations and isolates without mutations. We found significant differences between the wildtype genes and isolates genes with mutations *mtrF*_V213I (higher expression), *gyrA*_S91F (lower), *gyrA*_D95G (lower), *parC*_S87N (lower), *parC*_V384I (higher), and *macA*_A8S (lower). A combination of mutations and other factors contribute to increased MICs; however, in our cohort, the majority of resistance to penicillin and tetracycline is attributed to *bla*_*TEM*_ and *tetM*, respectively [35].

It was previously reported that *N. gonorrhoeae* transcriptional responses to infection differed in genital specimens from men and women, and AMR gene expression was increased in men, with a higher expression of MtrCDE efflux pump-related genes, suggesting that the expression of AMR genes is driven by sex-specific environments [49]. While overall gene expression signatures may be sex-specific, we found that in our cohort of South African patients, there were no significant differences in expression of resistance-associated targets between isolates from men and women. In addition, the *farA* and *farB* genes which encode the FarAB efflux pump (export host-derived antimicrobials, including cationic antimicrobial peptides and long-chain fatty acids) [4], were similarly expressed. Based on this outcome, we found that while therapeutic strategies could be based on gender, when using a diagnostic assay, there was no need to streamline the gene target profile based on gender, and that the same targets can be used for AMR detection for specimens from males and females in our setting.

In this study, we determined thresholds of mRNA levels, which could be used for resistance prediction within a South African population. Using regression analysis, we then determined the strongest predictors associated with AMR status. *N. gonorrhoeae* AMR is associated with numerous resistance mechanisms. We found the most significant markers for AMR status prediction in this population to be *mtrC*, *gyrA, parE, rpsJ,* and 23S rRNA. Our approach provides thresholds with high sensitivity for each of the strongest predictors using ROC analysis and can be used as a rule-in test for resistance prediction.

A limitation of the study and proposed models was the lack of clinical isolates with resistance to spectinomycin, azithromycin, cefixime, and ceftriaxone. Future studies will include a larger data set. Another limitation is that this needs to be evaluated on clinical specimens direct from patients to establish sensitivity as well as time-to-result. Also, the strongest predictors for the antibiotic resistance detection were limited to the isolates data used to generate the regression equations and is valid for the local setting in which these strains were isolated. This enforces the need for continuous local surveillance of isolates. Similar regression analysis can be used to identify candidate markers for resistance prediction in different geographic areas.

## 5. Conclusion

Using real-time qPCR, we have identified that mRNA levels of potential candidate markers of resistance can be used for AMR testing of *N. gonorrhoeae*. Together with the ROC cutoff values, these can be explored further as a set of genetic markers of antimicrobial resistance in our setting. A larger-scale validation is required, and evaluation directly from clinical specimens. Identifying local candidate markers has the potential to be used as a near-patient test in addition to NAATs identification.

## Figures and Tables

**Table 1:** Primers used for real-time qPCR.

Gene	Locus tag	Primer sequence (5′-3′)	Amplicon size (bp)	Description
*penA*	NGO1542	penAF_ACCGAAAGACATCGTCGCCT	172	Penicillin-binding protein
		penAR_CGTCGGCACAAGCAAACTGT		
*ponA*	NGO0099	ponAF_GGAGTGGGTCTGGTTGCCAT	201	Penicillin-binding protein 1A
		ponAR_GGCAATAACCGCATTCCGCA		
*pilQ*	NGO0094	pilQF_ACGAGGCTTTGGATTGCGAG	234	Type IV pilus secretin PilQ.
		pilQR_TTATGCTTTTTGCCGCGACCG		
*rpsJ*	NGO1841	rpsJF_CCATCAGGCGCAAATGGGTG	179	30S ribosomal protein S10
		rpsJR_CGCCCTGATTGACCGTTCTG		
16S rRNA	NGO_r03	16SrRNAF_AGCCGTAACACAGGTGCTGC	209	16S ribosomal RNA
	NGO_r06			
	NGO_r09	16S rRNAR_GACCATTGTATGACGTGTGAAGCC		
	NGO_r12			
*gyrA*	NGO0629	gyrAF_TTGTGAGAAGCTGGATGACGG	185	DNA gyrase subunit A
		gyrAR_TGGACGAAGGCGAAACCTTG		
*parC*	NGO1259	parCF_GGTTGCCGTCTATGCCTCCT	213	DNA topoisomerase IV subunit A
		parCR_CGCCTGCCTTCGCTTTCAAT		
*parE*	NGO1333	parEF_GCCTTCGCGTTCCATCCAAG	166	DNA topoisomerase IV subunit B
		parER_GATGAACCCCGACCAGCTCA		
23S rRNA	NGO_r02	23SF_TGCTTCCAAGCCTTCCAC	171	23S ribosomal RNA
	NGO_r05	23SR_GAATGGCGTAACGATGGC		
	NGO_r08			
	NGO_r11			
*mtrR*	NGO1366	mtrRF_CGTTGGACGGGCTGATTTGG	118	HTH-type transcriptional regulator MtrR
		mtrRR_CGCAGGCAGGGATGGTTTTC		
*mtrA*	NGO1250	mtrAF_GTGCCTTTTGGGCGGACAAT	173	Transcriptional activator of mtrCDE
		mtrAR_TCCGTCGTGGCTCAACACAT		
*mtrC*	NGO1365	mtrCF_TCCACAACCACCTTGTCCCC	136	Cation/multidrug efflux protein
		mtrCR_GCGGTGCGAAAGATACCGTG		
*mtrD*	NGO1364	mtrDF_CGTATTGCTGGACGGTTGCC	242	Cation/multidrug efflux protein
		mtrDR_GCACGCCATTTATCCGGGTG		
*mtrE*	NGO1363	mtrEF_AGACGGCATTTGTTTGCCCG	165	Multidrug transporter
		mtrER_ATTTGCTCGATGCGGAACGC		
*mtrF*	NGO1368	mtrFF_ACAGTCGAATGGCTGGGCAA	99	Integral membrane protein. Newly described efflux pump
		mtrFR_GAAATACGCACCGACGGCAG		
*macA*	NGO1440	macAF_TTCACGGTCAGCGACGGAAT	115	Macrolide transport protein MacA
		macAR_CCCGTTCGTTTGTGCCGAAT		
*macB*	NGO1439	macBF_ATCTGCCTGATGCTGTCGCT	199	Macrolide ABC transporter ATP-binding protein/permease
		macBR_CCGACGTGCTGATGCTTTGG		
*norM*	NGO0395	norMF_ATCGAAACGGTAGGCGAGCA	140	Multidrug efflux protein
		norMR_AACCGGCAGACTTCACCCAA		
*farA*	NGO1683	farAF_GCGGATTGCCCGAGGATTTC	183	Multidrug resistance protein
		farAR_GCTGAACCGCGAAGATGTGG		
*farB*	NGO1682	farBF_TGTTGCGGAATAGGGCGTGA	170	EmrB/QacA subfamily multidrug transporter
		farBR_CACTGTCGCACATGAAGGGC		

**Table 2:** Statistically significant logistic regression models of genes associated with AMR as determined by EUCAST MIC interpretation.

Drug	Model	Beta	Std. error	Wald	Df	Sig.
PEN	*penA*	−477.444	480.180	0.989	1	0.320
	*pilQ*	−45.427	409.199	0.012	1	0.912
	*ponA*	48.974	161.466	0.092	1	0.762
	*mtrR*	15.605	22.818	0.468	1	0.494
	*mtrC*	**−21.799**	**9.640**	**5.113**	**1**	**0.024**
	*mtrD*	40.099	207.809	0.037	1	0.847
	*mtrE*	38.026	94.177	0.163	1	0.686
	*mtrA*	6.592	7.251	0.827	1	0.363
	*mtrF*	7.687	13.078	0.345	1	0.557
	Constant	2.351	0.422	31.058	1	≤0.001

CIP	*gyrA*	**22.691**	**10.286**	**4.867**	**1**	**0.027**
	*parC*	92.723	155.843	0.354	1	0.552
	*parE*	**−457.577**	**218.360**	**4.391**	**1**	**0.036**
	*norM*	−95.040	97.960	0.941	1	0.332
	Constant	0.769	0.243	9.994	1	0.002

TET	*rpsJ*	**−1431.586**	**734.579**	**3.798**	**1**	**0.047**
	*mtrR*	−58.549	101.811	0.331	1	.565
	*mtrC*	−23.590	40.055	0.347	1	.556
	*mtrD*	415.541	449.943	0.853	1	.356
	*mtrE*	−179.181	257.407	0.485	1	.486
	*mtrA*	33.872	44.609	0.577	1	.448
	*mtrF*	24.805	38.481	0.415	1	.519
	Constant	4.886	1.182	17.075	1	≤.001

AZ	**23S**	0.212	0.104	4.140	1	0.042
	*macA*	−104.209	68.197	2.335	1	0.126
	*macB*	429.998	317.542	1.834	1	0.176
	*mtrR*	−51.528	171.359	.090	1	0.764
	*mtrC*	43.816	30.898	2.011	1	0.156
	*mtrD*	−767.506	1316.124	.340	1	0.560
	*mtrE*	−2304.401	1446.997	2.536	1	0.111
	*mtrA*	−47.677	65.918	.523	1	0.470
	*mtrF*	162.321	126.611	1.644	1	0.200
	Constant	−3.426	1.545	4.916	1	0.027

Bold = significant resistance-associated marker for prediction of antimicrobial resistance in this setting. *∗* The regression models for spectinomycin and ESC were not statistically significant due to a low number of data points in the resistant group, and therefore not included in this table.

**Table 3:** Diagnostic performance of AMR-associated genes to detect antibiotic resistance using cutoff values determined by ROC analysis.

Drug	Gene	AUC	*p* value	Cutoff	Sens %	95% CI	Spec %	95% CI	Likelihood ratio
PEN	*penA*	0.63	0.027	<0.0018	84	74 to 91	38	21 to 56	1.35
	*ponA*	0.63	0.037	<0.0021	82	72 to 89	36	19 to 56	1.27
	*mtrR*	0.62	0.052	<0.0137	80	70 to 88	40	23 to 59	1.33
	***mtrC***	0.63	0.027	<0.0890	84	75 to 91	44	26 to 62	1.49
	*mtrD*	0.63	0.033	<0.0025	83	74 to 90	41	24 to 59	1.4
	*mtrE*	0.65	0.017	<0.0081	84	75 to 91	34	18 to 54	1.28
	*mtrA*	0.62	0.039	<0.1003	86	77 to 93	25	11 to 43	1.15

CIP	***gyrA***	0.66	0.003	<0.0518	83	73 to 91	33	20 to 48	1.24
	*parC*	0.65	0.004	<0.0032	85	74 to 92	29	17 to 43	1.19
	***parE***	0.67	0.001	<0.0033	88	78 to 94	33	20 to 48	1.3
	*norM*	0.65	0.006	<0.0084	93	85 to 98	29	17 to 43	1.3

TET	***rpsJ***	0.72	0.068	<0.0012	91	84 to 96	33	4 to 78	1.37
	*mtrR*	0.71	0.040	<0.0431	96	91 to 99	33	7 to 70	1.45
	*mtrC*	0.76	0.009	<0.1376	90	83 to 95	44	14 to 79	1.62
	*mtrA*	0.72	0.027	<0.1024	87	80 to 93	44	1 to 79	1.57
	*mtrF*	0.75	0.020	<0.1217	97	92 to 99	38	9 to 76	1.56
SPT	16S	0.96	0.115	<0.0454	100	2.5 to 100	96	91 to 99	24

AZ	**23S**	0.99	≤0.001	>7.7540	86	42 to 99	99	95 to 99	92.57
	*macA*	0.86	0.003	<0.0628	100	54 to 100	42	32 to 51	1.71
	*macB*	0.78	0.013	<0.0281	100	5 to 100	31	22 to 40	1.44
	*mtrR*	0.78	0.011	<0.0035	86	42 to 100	60	51 to 69	2.15
	*mtrC*	0.69	0.094	<0.0435	86	42 to 100	47	37 to 57	1.61
	*mtrD*	0.83	0.003	<0.0010	86	42 to 100	47	37 to 57	1.61
	*mtrE*	0.81	0.006	<0.0011	86	42 to 100	68	58 to 76	2.67
	*mtrA*	0.80	0.007	<0.0146	86	42 to 100	72	62 to 80	3.03
	*mtrF*	0.80	0.009	<0.0070	86	42 to 100	64	5 to 73	2.36

ESC	*penA*	1.0	0.086	<7.650*e *− 005	100	2.5 to 100	99	95 to 99	114
	*mtrR*	0.89	0.023	<0.0014	100	29 to 100	80	71 to 87	4.91
	*mtrC*	0.87	0.028	<0.0170	100	29 to 100	78	69 to 85	4.56
	*mtrD*	0.96	0.007	≤0.001	100	29 to 100	91	84 to 96	11.4
	*mtrE*	0.94	0.01	≤0.001	100	29 to 100	87	79 to 92	7.53
	*mtrA*	0.92	0.014	<0.0070	100	29 to 100	89	81 to 94	8.77
	*mtrF*	0.92	0.013	<0.0031	100	29 to 100	87	79 to 92	7.53

Bold = significant resistance-associated marker for prediction of antimicrobial resistance in this setting.

## Data Availability

Whole-genome sequence data is available in DDBJ/ENA/GenBank with the BioProject number PRJNA681740.

## Supplementary Materials

Supplementary Materials Supplementary Figure 1. Confirmation of *Neisseria gonorrhoeae* isolates using real-time PCR. Supplementary Figure 2. Antibiotic susceptibility profile of *Neisseria gonorrhoeae* isolated from South Africa between 2013 and 2017. Supplementary Table 1. Results of presumptive and confirmatory identification tests for *Neisseria gonorrhoeae.* Supplementary Table 2. Comparison of mRNA expression levels between antibiotic susceptible and nonsusceptible *N. gonorrhoeae* isolates from South Africa. Supplementary Table 3. Comparison of mRNA expression levels between South African *N. gonorrhoeae* isolates with mutations and with no mutations.
