# Supplementary material for: Assessment of Antibiotic Resistance and Efflux Pump Gene Expression in Neisseria Gonorrhoeae Isolates from South Africa by Quantitative Real-Time PCR and Regression Analysis
Source: Int J Microbiol. 2022 Oct 21;2022:7318325. doi: 10.1155/2022/7318325 (PMC9616671; doi:10.1155/2022/7318325)
Supplement: Supplementary Materials — Supplementary Figure 1. Confirmation of Neisseria gonorrhoeae isolates using real-time PCR. Supplementary Figure 2. Antibiotic susceptibility profile of Neisseria gonorrhoeae isolated from South Africa between 2013 and 2017. Supplementary Table 1. Results of presumptive and confirmatory identification tests for Neisseria gonorrhoeae. Supplementary Table 2. Comparison of mRNA expression levels between antibiotic susceptible and nonsusceptible N. gonorrhoeae isolates from South Africa. Supplementary Table 3. Comparison of mRNA expression levels between South African N. gonorrhoeae isolates with mutations and with no mutations. [file 7318325.f1.docx]

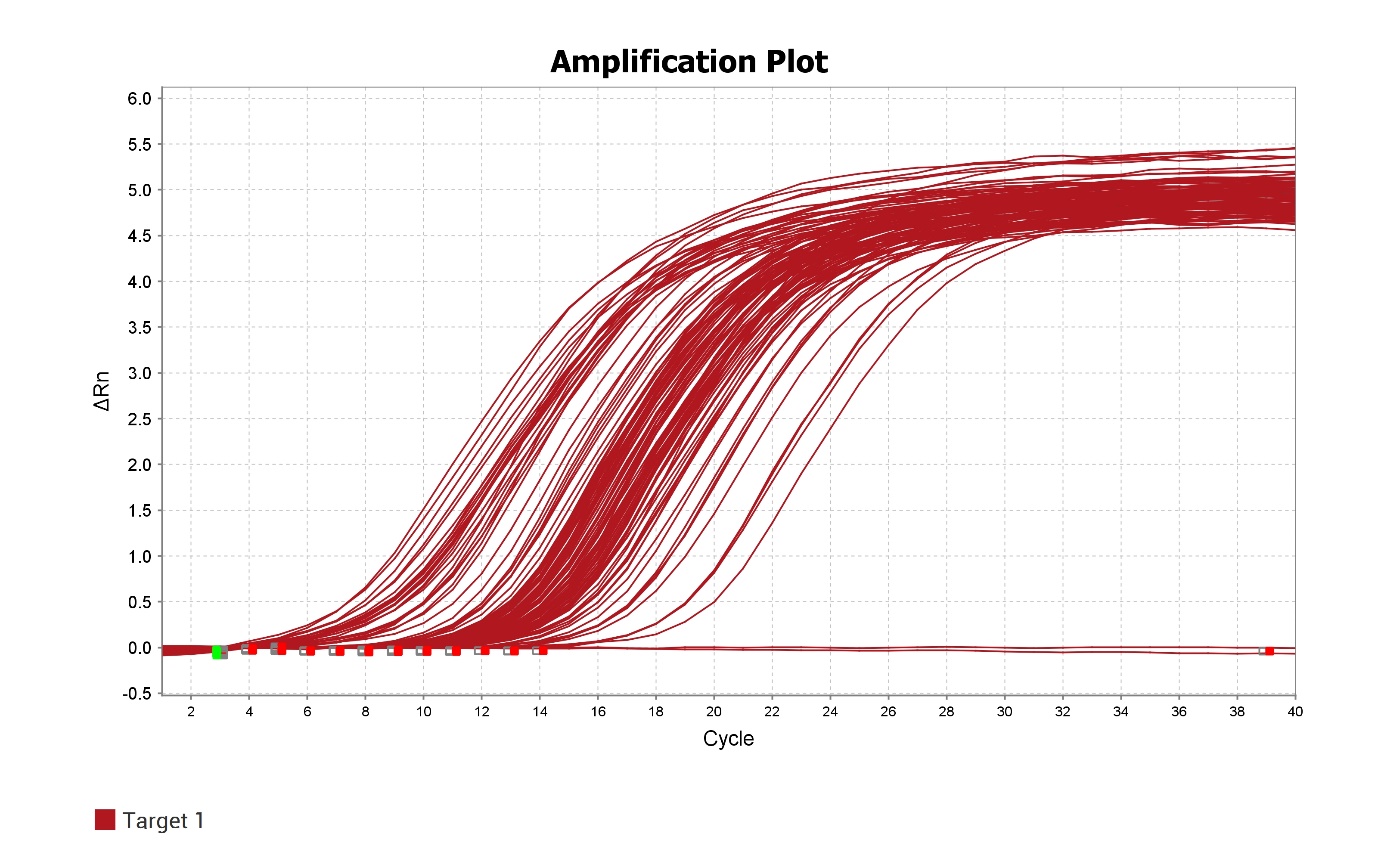


**Figure 1.** Confirmation of *Neisseria gonorrhoeae* isolates using real-time PCR


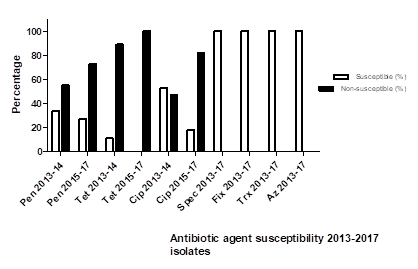


**Figure 2.** Antibiotic susceptibility profile of *Neisseria gonorrhoeae* isolated from South Africa between 2013 - 2017

**Table 1.** Results of Presumptive and confirmatory identification tests for *Neisseria gonorrhoeae*

|  | **Grams stain** | **Rapid Oxidase test** | **Phadebact® Monoclonal GC test** | **Molecular ID** |
| --- | --- | --- | --- | --- |
| **Positive control** | *Staph. aureus* | *Ps. aeruginosa* | QC as per kit | *N. gonorrhoeae* positive |
| **Negative control** | *E. coli* | *E. coli* | QC as per kit | No template control |
| **Isolate** |  |  |  |  |
| 1 | Gram negative diplococci | Positive | Positive | Positive |
| 2 | Gram negative diplococci | Positive | Positive | Positive |
| 3 | Gram negative diplococci | Positive | Positive | Positive |
| 4 | Gram negative diplococci | Positive | Positive | Positive |
| 5 | Gram negative diplococci | Positive | Positive | Positive |
| 6 | Gram negative diplococci | Positive | Positive | Positive |
| 7 | Gram negative diplococci | Positive | Positive | Positive |
| 8 | Gram negative diplococci | Positive | Positive | Positive |
| 9 | Gram negative diplococci | Positive | Positive | Positive |
| 10 | Gram negative diplococci | Positive | Positive | Positive |
| 11 | Gram negative diplococci | Positive | Positive | Positive |
| 12 | Gram negative diplococci | Positive | Positive | Positive |
| 13 | Gram negative diplococci | Positive | Positive | Positive |
| 14 | Gram negative diplococci | Positive | Positive | Positive |
| 15 | Gram negative diplococci | Positive | Positive | Positive |
| 16 | Gram negative diplococci | Positive | Positive | Positive |
| 17 | Gram negative diplococci | Positive | Positive | Positive |
| 18 | Gram negative diplococci | Positive | Positive | Positive |
| 19 | Gram negative diplococci | Positive | Positive | Positive |
| 20 | Gram negative diplococci | Positive | Positive | Positive |
| 21 | Gram negative diplococci | Positive | Positive | Positive |
| 22 | Gram negative diplococci | Positive | Positive | Positive |
| 23 | Gram negative diplococci | Positive | Positive | Positive |
| 24 | Gram negative diplococci | Positive | Positive | Positive |
| 25 | Gram negative diplococci | Positive | Positive | Positive |
| 26 | Gram negative diplococci | Positive | Positive | Positive |
| 27 | Gram negative diplococci | Positive | Positive | Positive |
| 28 | Gram negative diplococci | Positive | Positive | Positive |
| 29 | Gram negative diplococci | Positive | Positive | Positive |
| 30 | Gram negative diplococci | Positive | Positive | Positive |
| 31 | Gram negative diplococci | Positive | Positive | Positive |
| 32 | Gram negative diplococci | Positive | Positive | Positive |
| 33 | Gram negative diplococci | Positive | Positive | Positive |
| 34 | Gram negative diplococci | Positive | Positive | Positive |
| 35 | Gram negative diplococci | Positive | Positive | Positive |
| 36 | Gram negative diplococci | Positive | Positive | Positive |
| 37 | Gram negative diplococci | Positive | Positive | Positive |
| 38 | Gram negative diplococci | Positive | Positive | Positive |
| 39 | Gram negative diplococci | Positive | Positive | Positive |
| 40 | Gram negative diplococci | Positive | Positive | Positive |
| 41 | Gram negative diplococci | Positive | Positive | Positive |
| 42 | Gram negative diplococci | Positive | Positive | Positive |
| 43 | Gram negative diplococci | Positive | Positive | Positive |
| 44 | Gram negative diplococci | Positive | Positive | Positive |
| 45 | Gram negative diplococci | Positive | Positive | Positive |
| 46 | Gram negative diplococci | Positive | Positive | Positive |
| 47 | Gram negative diplococci | Positive | Positive | Positive |
| 48 | Gram negative diplococci | Positive | Positive | Positive |
| 49 | Gram negative diplococci | Positive | Positive | Positive |
| 50 | Gram negative diplococci | Positive | Positive | Positive |
| 51 | Gram negative diplococci | Positive | Positive | Positive |
| 52 | Gram negative diplococci | Positive | Positive | Positive |
| 53 | Gram negative diplococci | Positive | Positive | Positive |
| 54 | Gram negative diplococci | Positive | Positive | Positive |
| 55 | Gram negative diplococci | Positive | Positive | Positive |
| 56 | Gram negative diplococci | Positive | Positive | Positive |
| 57 | Gram negative diplococci | Positive | Positive | Positive |
| 58 | Gram negative diplococci | Positive | Positive | Positive |
| 59 | Gram negative diplococci | Positive | Positive | Positive |
| 60 | Gram negative diplococci | Positive | Positive | Positive |
| 61 | Gram negative diplococci | Positive | Positive | Positive |
| 62 | Gram negative diplococci | Positive | Positive | Positive |
| 63 | Gram negative diplococci | Positive | Positive | Positive |
| 64 | Gram negative diplococci | Positive | Positive | Positive |
| 65 | Gram negative diplococci | Positive | Positive | Positive |
| 66 | Gram negative diplococci | Positive | Positive | Positive |
| 67 | Gram negative diplococci | Positive | Positive | Positive |
| 68 | Gram negative diplococci | Positive | Positive | Positive |
| 69 | Gram negative diplococci | Positive | Positive | Positive |
| 70 | Gram negative diplococci | Positive | Positive | Positive |
| 71 | Gram negative diplococci | Positive | Positive | Positive |
| 72 | Gram negative diplococci | Positive | Positive | Positive |
| 73 | Gram negative diplococci | Positive | Positive | Positive |
| 74 | Gram negative diplococci | Positive | Positive | Positive |
| 75 | Gram negative diplococci | Positive | Positive | Positive |
| 76 | Gram negative diplococci | Positive | Positive | Positive |
| 77 | Gram negative diplococci | Positive | Positive | Positive |
| 78 | Gram negative diplococci | Positive | Positive | Positive |
| 79 | Gram negative diplococci | Positive | Positive | Positive |
| 80 | Gram negative diplococci | Positive | Positive | Positive |
| 81 | Gram negative diplococci | Positive | Positive | Positive |
| 82 | Gram negative diplococci | Positive | Positive | Positive |
| 83 | Gram negative diplococci | Positive | Positive | Positive |
| 84 | Gram negative diplococci | Positive | Positive | Positive |
| 85 | Gram negative diplococci | Positive | Positive | Positive |
| 86 | Gram negative diplococci | Positive | Positive | Positive |
| 87 | Gram negative diplococci | Positive | Positive | Positive |
| 88 | Gram negative diplococci | Positive | Positive | Positive |
| 89 | Gram negative diplococci | Positive | Positive | Positive |
| 90 | Gram negative diplococci | Positive | Positive | Positive |
| 91 | Gram negative diplococci | Positive | Positive | Positive |
| 92 | Gram negative diplococci | Positive | Positive | Positive |
| 93 | Gram negative diplococci | Positive | Positive | Positive |
| 94 | Gram negative diplococci | Positive | Positive | Positive |
| 95 | Gram negative diplococci | Positive | Positive | Positive |
| 96 | Gram negative diplococci | Positive | Positive | Positive |
| 97 | Gram negative diplococci | Positive | Positive | Positive |
| 98 | Gram negative diplococci | Positive | Positive | Positive |
| 99 | Gram negative diplococci | Positive | Positive | Positive |
| 100 | Gram negative diplococci | Positive | Positive | Positive |
| 101 | Gram negative diplococci | Positive | Positive | Positive |
| 102 | Gram negative diplococci | Positive | Positive | Positive |
| 103 | Gram negative diplococci | Positive | Positive | Positive |
| 104 | Gram negative diplococci | Positive | Positive | Positive |
| 105 | Gram negative diplococci | Positive | Positive | Positive |
| 106 | Gram negative diplococci | Positive | Positive | Positive |
| 107 | Gram negative diplococci | Positive | Positive | Positive |
| 108 | Gram negative diplococci | Positive | Positive | Positive |
| 109 | Gram negative diplococci | Positive | Positive | Positive |
| 110 | Gram negative diplococci | Positive | Positive | Positive |

**Table 2.** Comparison of mRNA expression levels between antibiotic susceptible and non-susceptible *N. gonorrhoeae* isolates from South Africa

|  |  |  | Maximum mRNA levels | |
| --- | --- | --- | --- | --- |
| **Drug** | **Molecular marker** | ***p*-value** | Susceptible | Non-susceptible |
| **Penicillin** |  |  |  |  |
|  | *penA* | 0.0179 | .013995368965410 | .005405208474222 |
|  | *ponA* | 0.0175 | .016802524150904 | .013304414699338 |
|  | *pilQ* | 0.041 | .004629049028532 | .010023578600167 |
|  | *mtrR* | 0.025 | .422258661676569 | .123580764675083 |
|  | *mtrC* | 0.011 | .866051739398912 | .367878932702907 |
|  | *mtrD* | 0.031 | .026055846868547 | .022800915505086 |
|  | *mtrE* | 0.0149 | .059569209208308 | .074138041381467 |
|  | *mtrA* | 0.032 | .804628425157153 | .492365334923846 |
|  | *mtrF* | 0.17 | .663436474558126 | .359262346941044 |
| **Ciprofloxacin** |  |  |  |  |
|  | *gyrA* | 0.001 | 1.815683377645425 | .336686166356001 |
|  | *parC* | 0.0009 | .092289137317466 | .015024203050745 |
|  | *parE* | 0.0003 | .135396980478781 | .013320355524183 |
|  | *norM* | 0.001 | .187527488718341 | .033929760170589 |
| **Tetracycline** |  |  |  |  |
|  | *rpsJ* | 0.028 | .003079531254864 | .003340843610938 |
|  | *mtrR* | 0.020 | .153528975619493 | .422258661676569 |
|  | *mtrC* | 0.001 | .497830303135074 | .367878932702907 |
|  | *mtrD* | 0.045 | .026055846868547 | .009394701404530 |
|  | *mtrE* | 0.024 | .045347824830046 | .059569209208308 |
|  | *mtrA* | 0.009 | .804628425157153 | .466597321357186 |
|  | *mtrF* | 0.003 | .354199825063687 | 1.264369840151505 |
| **Azithromycin** |  |  |  |  |
|  | 23S | <0.0001 | 36.331163946965800 | 52.107068374993810 |
|  | *macA* | 0.0005 | 3.823937662296675 | .062504510133588 |
|  | *macB* | 0.013 | 2.110264098527542 | .027639796167259 |
|  | *mtrR* | 0.0169 | .898054697137212 | .007305889397900 |
|  | *mtrC* | 0.1116 | 4.611681720362236 | .058976093385084 |
|  | *mtrD* | 0.005 | .167094059491162 | .001391966675982 |
|  | *mtrE* | 0.003 | .332291732604477 | .004839743289964 |
|  | *mtrA* | 0.009 | 2.423207674210328 | .042630412315636 |
|  | *mtrF* | 0.0107 | 1.264369840151505 | .025486631764335 |
| **Spectinomycin** |  |  |  |  |
|  | 16S | 0.0007 |  |  |
| **ESC** |  |  |  |  |
|  | *penA* | <0.0001 | .199556812734944 | .000000066567092 |
|  | *mtrR* | 0.028 | .898054697137212 | .001356907659756 |
|  | *mtrC* | 0.056 | 4.611681720362236 | .016415439723273 |
|  | *mtrD* | 0.005 | .026055846868547 | .000295260367481 |
|  | *mtrE* | 0.005 | .074138041381467 | .000283319866110 |
|  | *mtrA* | 0.008 | .804628425157153 | .006955403158443 |
|  | *mtrF* | 0.026 | .663436474558126 | .003098536015322 |

**Table 3.** Comparison of mRNA expression levels between South African *N. gonorrhoeae* isolates with mutations and with no mutations

|  |  |  |  |  |
| --- | --- | --- | --- | --- |
| **Drug** | **Molecular marker** | ***p*-value** | **Mutation** | ***p*-value** |
| **Penicillin** |  |  |  |  |
|  | *penA* | 0.0179 | Y201H | 0.006 |
|  |  |  | F505L | 0.017 |
|  |  |  | A511V | 0.017 |
|  |  |  | A517G | 0.017 |
|  |  |  | H542N | 0.231 |
|  |  |  | P522L | 0.512 |
|  |  |  | P553V | 0.07 |
|  |  |  | K556Q | 0.07 |
|  |  |  | I557V | 0.07 |
|  |  |  | I567V | 0.047 |
|  |  |  | A575N | 0.047 |
|  |  |  | A576V | 0.047 |
|  |  |  | 578insA | 0.047 |
|  | *ponA* | 0.0175 | L421P | 0.73 |
|  |  |  | A375T | 0.44 |
|  |  |  | K362E | 0.33 |
|  | *pilQ* | 0.041 | Del151 | 0.018 |
|  |  |  | S132P | 0.089 |
|  |  |  | Ins176 | 0.18 |
|  |  |  | S341N | 0.87 |
|  |  |  | T502K | 0.60 |
|  |  |  | G503D | 0.66 |
|  |  |  | T511M | 0.21 |
|  |  |  | G517S | 0.07 |
|  |  |  | G525D | 0.33 |
|  |  |  | D526N | 0.36 |
|  |  |  | E529G | 0.36 |
|  |  |  | A530V | 0.76 |
|  |  |  | T532N | 0.07 |
|  |  |  | A539V | 0.32 |
|  |  |  | N648S | 0.31 |
|  | *mtrR* | 0.025 | A39T | 0.91 |
|  |  |  | del41-44 | 0.14 |
|  |  |  | G45D | 0.44 |
|  |  |  | D79N | 0.70 |
|  |  |  | T86A | 0.65 |
|  |  |  | H105Y | 0.63 |
|  |  |  | H118E | 0.03 |
|  |  |  | T119Q | 0.03 |
|  |  |  | E120N | 0.03 |
|  |  |  | Q121A | 0.03 |
|  |  |  | N122A | 0.03 |
|  |  |  | A123V | 0.03 |
|  |  |  | A124I | 0.03 |
|  |  |  | V125A | 0.03 |
|  |  |  | I126H | 0.03 |
|  |  |  | A127T | 0.03 |
|  | *mtrC* | 0.011 | G163S | 0.01 |
|  |  |  | E275D | All |
|  |  |  | P387S | 0.01 |
|  | *mtrD* | 0.031 | I397T | 0.3 |
|  |  |  | A489V | 0.67 |
|  |  |  | A577V | 0.97 |
|  |  |  | T653A | 0.67 |
|  |  |  | S661A | All |
|  |  |  | D720E | 0.1 |
|  |  |  | P820S | 0.53 |
|  |  |  | V1020I | 0.53 |
|  | *mtrE* | 0.0149 | I47V | 0.98 |
|  |  |  | K181E | All |
|  |  |  | F335L | 0.09 |
|  |  |  | A383V | 0.007 |
|  |  |  | I429S | 0.77 |
|  | *mtrA* | 0.032 | No mut |  |
|  | *mtrF* | 0.17 | I192L | 0.9 |
|  |  |  | V213I | 0.027 |
|  |  |  | A223V | 0.91 |
|  |  |  | A224T | 0.91 |
|  |  |  | V281I | 0.43 |
|  |  |  | I333V | 0.53 |
|  |  |  | G394A | 0.66 |
|  |  |  | I470M | 0.34 |
|  |  |  | T515A | All |
|  |  |  | F518L | 0.33 |
|  |  |  | V521A | 0.15 |
| **Ciprofloxacin** |  |  |  |  |
|  | *gyrA* | 0.001 | S91F | 0.002 |
|  |  |  | D95G | 0.026 |
|  |  |  | D95A | 0.327 |
|  |  |  | A70V | 0.62 |
|  |  |  | I250M | 0.78 |
|  |  |  | D704N | 0.29 |
|  |  |  | G779S | 0.166 |
|  | *parC* | 0.0009 | D86N | 0.719 |
|  |  |  | S87N | 0.023 |
|  |  |  | S87I | 0.48 |
|  |  |  | E91Q | 0.41 |
|  |  |  | E91G | 0.09 |
|  |  |  | V384I | 0.0007 |
|  |  |  | A156T | 0.71 |
|  |  |  | E378K | 0.48 |
|  |  |  | V435A | 0.86 |
|  |  |  | F479L | 0.40 |
|  |  |  | V596I | 0.44 |
|  |  |  | E725K | 0.05 |
|  | *parE* | 0.0003 | V89A | 0.91 |
|  |  |  | T234A | 0.82 |
|  |  |  | A412T | 0.06 |
|  |  |  | P456S | 0.9 |
|  |  |  | A645T | 0.9 |
|  |  |  | V485I | All |
|  | *norM* | 0.001 | M295I | 0.184 |
|  |  |  | K457E | 0.721 |
| **Tetracycline** |  |  |  |  |
|  | *rpsJ* | 0.028 | V57M | 0.81 |
|  | *mtrR* | 0.020 | A39T | 0.76 |
|  |  |  | del41-44 | 0.20 |
|  |  |  | G45D | 0.52 |
|  |  |  | D79N | 0.89 |
|  |  |  | T86A | 0.51 |
|  |  |  | H105Y | 0.42 |
|  |  |  | H118E | 0.06 |
|  |  |  | T119Q | 0.06 |
|  |  |  | E120N | 0.06 |
|  |  |  | Q121A | 0.06 |
|  |  |  | N122A | 0.06 |
|  |  |  | A123V | 0.06 |
|  |  |  | A124I | 0.06 |
|  |  |  | V125A | 0.06 |
|  |  |  | I126H | 0.06 |
|  |  |  | A127T | 0.06 |
|  | *mtrC* | 0.001 | G163S | 0.01 |
|  |  |  | E275D | All |
|  |  |  | P387S | 0.01 |
|  | *mtrD* | 0.045 | I397T | 0.39 |
|  |  |  | A489V | 0.69 |
|  |  |  | A577V | 0.99 |
|  |  |  | T653A | 0.69 |
|  |  |  | S661A | All |
|  |  |  | D720E | 0.10 |
|  |  |  | P820S | 0.50 |
|  |  |  | V1020I | 0.50 |
|  | *mtrE* | 0.024 | I47V | 0.50 |
|  |  |  | K181E | All |
|  |  |  | F335L | 0.08 |
|  |  |  | A383V | 0.007 |
|  |  |  | I429S | 0.99 |
|  | *mtrA* | 0.009 | No mut |  |
|  | *mtrF* | 0.003 | I192L | 0.90 |
|  |  |  | V213I | 0.02 |
|  |  |  | A223V | 0.91 |
|  |  |  | A224T | 0.91 |
|  |  |  | V281I | 0.43 |
|  |  |  | I333V | 0.53 |
|  |  |  | G394A | 0.66 |
|  |  |  | I470M | 0.34 |
|  |  |  | T515A | All |
|  |  |  | F518L | 0.33 |
|  |  |  | V521A | 0.15 |
| **Azithromycin** |  |  |  |  |
|  | 23S | <0.0001 | No mut |  |
|  | *macA* | 0.0005 | A8S | 0.01 |
|  |  |  | S23H | 0.22 |
|  |  |  | S23F | 0.12 |
|  |  |  | A32S | 0.62 |
|  |  |  | T37A | 0.42 |
|  |  |  | D42G | 0.67 |
|  |  |  | A63T | 0.06 |
|  |  |  | I98L | 0.18 |
|  |  |  | D99N | 0.99 |
|  |  |  | A130T | 0.78 |
|  |  |  | A139S | 0.20 |
|  |  |  | T208A | 0.20 |
|  |  |  | V342A | 0.61 |
|  |  |  | K351R | 0.98 |
|  |  |  | I373M | 0.12 |
|  |  |  | A385V | 0.44 |
|  |  |  | L386M | 0.46 |
|  |  |  | Del392 | 0.14 |
|  | *macB* | 0.013 | C12Y | 0.50 |
|  |  |  | R239Q | 0.82 |
|  |  |  | T346M | 0.55 |
|  | *mtrR* | 0.0169 | A39T | 0.71 |
|  |  |  | del41-44 | 0.20 |
|  |  |  | G45D | 0.52 |
|  |  |  | D79N | 0.99 |
|  |  |  | T86A | 0.44 |
|  |  |  | H105Y | 0.42 |
|  |  |  | H118E | 0.06 |
|  |  |  | T119Q | 0.06 |
|  |  |  | E120N | 0.06 |
|  |  |  | Q121A | 0.06 |
|  |  |  | N122A | 0.06 |
|  |  |  | A123V | 0.06 |
|  |  |  | A124I | 0.06 |
|  |  |  | V125A | 0.06 |
|  |  |  | I126H | 0.06 |
|  |  |  | A127T | 0.06 |
|  | *mtrC* | 0.1116 | G163S | 0.01 |
|  |  |  | E275D | All |
|  |  |  | P387S | 0.01 |
|  | *mtrD* | 0.005 | I397T | 0.30 |
|  |  |  | A489V | 0.67 |
|  |  |  | A577V | 0.97 |
|  |  |  | T653A | 0.67 |
|  |  |  | S661A | All |
|  |  |  | D720E | 0.10 |
|  |  |  | P820S | 0.53 |
|  |  |  | V1020I | 0.53 |
|  | *mtrE* | 0.003 | I47V | 0.52 |
|  |  |  | K181E | All |
|  |  |  | F335L | 0.08 |
|  |  |  | A383V | 0.007 |
|  |  |  | I429S | 0.98 |
|  | *mtrA* | 0.009 | No mut |  |
|  | *mtrF* | 0.0107 | I192L | 0.90 |
|  |  |  | V213I | 0.02 |
|  |  |  | A223V | 0.91 |
|  |  |  | A224T | 0.91 |
|  |  |  | V281I | 0.43 |
|  |  |  | I333V | 0.53 |
|  |  |  | G394A | 0.66 |
|  |  |  | I470M | 0.34 |
|  |  |  | T515A | All |
|  |  |  | F518L | 0.33 |
|  |  |  | V521A | 0.15 |
| **Spectinomycin** |  |  |  |  |
|  | 16S | 0.0007 | No mut |  |
| **ESC** |  |  |  |  |
|  | *penA* | <0.0001 | Y201H | 0.006 |
|  |  |  | F505L | 0.01 |
|  |  |  | A511V | 0.01 |
|  |  |  | A517G | 0.01 |
|  |  |  | H542N | 0.23 |
|  |  |  | P522L | 0.51 |
|  |  |  | P553V | 0.07 |
|  |  |  | K556Q | 0.07 |
|  |  |  | I557V | 0.07 |
|  |  |  | I567V | 0.04 |
|  |  |  | A575N | 0.04 |
|  |  |  | A576V | 0.04 |
|  |  |  | 578insA | 0.04 |
|  | *mtrR* | 0.028 | A39T | 0.75 |
|  |  |  | del41-44 | 0.20 |
|  |  |  | G45D | 0.52 |
|  |  |  | D79N | 0.93 |
|  |  |  | T86A | 0.48 |
|  |  |  | H105Y | 0.42 |
|  |  |  | H118E | 0.06 |
|  |  |  | T119Q | 0.06 |
|  |  |  | E120N | 0.06 |
|  |  |  | Q121A | 0.06 |
|  |  |  | N122A | 0.06 |
|  |  |  | A123V | 0.06 |
|  |  |  | A124I | 0.06 |
|  |  |  | V125A | 0.06 |
|  |  |  | I126H | 0.06 |
|  |  |  | A127T | 0.06 |
|  | *mtrC* | 0.056 | G163S | 0.01 |
|  |  |  | E275D | All |
|  |  |  | P387S | 0.01 |
|  | *mtrD* | 0.005 | I397T | 0.30 |
|  |  |  | A489V | 0.67 |
|  |  |  | A577V | 0.97 |
|  |  |  | T653A | 0.67 |
|  |  |  | S661A | All |
|  |  |  | D720E | 0.10 |
|  |  |  | P820S | 0.53 |
|  |  |  | V1020I | 0.53 |
|  | *mtrE* | 0.005 | I47V | 0.79 |
|  |  |  | K181E | All |
|  |  |  | F335L | 0.07 |
|  |  |  | A383V | 0.005 |
|  |  |  | I429S | 0.62 |
|  | *mtrA* | 0.008 | No mut |  |
|  | *mtrF* | 0.026 | I192L | 0.90 |
|  |  |  | V213I | 0.02 |
|  |  |  | A223V | 0.91 |
|  |  |  | A224T | 0.91 |
|  |  |  | V281I | 0.43 |
|  |  |  | I333V | 0.53 |
|  |  |  | G394A | 0.66 |
|  |  |  | I470M | 0.34 |
|  |  |  | T515A | All |
|  |  |  | F518L | 0.33 |
|  |  |  | V521A | 0.15 |
